# Predicting opportunities for improvement in trauma care using machine learning: a retrospective registry-based study at a major trauma centre

**DOI:** 10.1136/bmjopen-2025-099624

**Published:** 2025-06-06

**Authors:** Jonatan Attergrim, Kelvin Szolnoky, Lovisa Strömmer, Olof Brattström, Gunilla Wihlke, Martin Jacobsson, Martin Gerdin Wärnberg

**Affiliations:** 1 Department of Global Public Health, Karolinska Institutet, Stockholm, Sweden; 2 Perioperative Medicine and Intensive Care, Karolinska University Hospital, Stockholm, Sweden; 3 Department of Medical Epidemiology and Biostatistics, Karolinska Institutet, Stockholm, Sweden; 4 Department of Surgery, Capio S:t Görans Hospital, Stockholm, Sweden; 5 Division of Surgery, Department of Clinical Science, Intervention and Technology (CLINTEC), Karolinska Institutet, Stockholm, Sweden; 6 Department of Anesthesiology, Mora Hospital, Mora, Sweden; 7 Trauma and Reparative Medicine, Karolinska University Hospital, Stockholm, Sweden; 8 Department of Biomedical Engineering and Health Systems, KTH Royal Institute of Technology, Stockholm, Sweden

**Keywords:** Trauma, Quality Improvement, Machine Learning

## Abstract

**Objective:**

To develop models to predict opportunities for improvement in trauma care and compare the performance of these models to the currently used audit filters.

**Design:**

Retrospective registry-based study.

**Setting:**

Single-centre, Scandinavian level one equivalent trauma centre.

**Participants:**

8220 adult trauma patients screened for opportunities for improvement between 2013 and 2022.

**Primary and secondary outcome measures:**

Two machine learning models (logistic regression and XGBoost) and the currently used audit filters were compared. Internal validation by an expanding window approach with annual updates was used for model evaluation. Performance measured by discrimination, calibration, sensitivity and false positive rate of opportunities for improvement prediction.

**Results:**

A total of 8220 patients, with a mean age of 45 years, were analysed; 69% were men with a mean injury severity score of 12. Opportunities for improvement were identified in 496 (6%) patients. Both the logistic regression and XGBoost models were well-calibrated, with intercalibration indices of 0.02 and 0.02, respectively. The models demonstrated higher areas under the receiver operating characteristic curve (AUCs) (logistic regression: 0.71; XGBoost: 0.74). The XGBoost model had a lower false positive rate at a similar sensitivity (false positive rate: 0.63). The audit filters had an AUC of 0.62 and a false positive rate of 0.67.

**Conclusions:**

The logistic regression and XGBoost models outperformed audit filters in predicting opportunities for improvement among adult trauma patients and can potentially be used to improve systems for selecting patients for trauma peer review.

**STRENGTHS AND LIMITATIONS OF THIS STUDY**

- The study employs machine learning models to predict opportunities for improvement in trauma care, offering a novel approach to screen for errors in patient care superior to traditional methods (audit filters).
- The inclusion of a year-by-year simulation of prospective implementation adds realism to the study design, reflecting potential challenges in real-world applications.
- The models were evaluated against opportunities for improvement identified by the current peer review system, which may result in false negatives due to limitations in the audit filter and review process.
- Opportunities for improvement were defined as a binary variable but encompass a heterogeneous set of outcomes, ranging from preventable deaths to communication errors, presenting a challenge for model prediction.

## Background

 Trauma is a leading cause of death and disability worldwide.^1 2^ Peer review of patient cases, sometimes referred to as performance improvement, is a critical component of trauma quality improvement programmes.^3^,^5^ This review ideally involves representatives from all disciplines and professions involved in trauma care to identify opportunities for improvement, which are preventable events in patient care with adverse outcomes.^6^

The current state-of-the-art systems for selecting patient cases for peer review use audit filters, sometimes in combination with individual human screening.^7^ Audit filters are sentinel events in patient care that are associated with suboptimal care and potentially poor patient outcomes, such as delays in key interventions or unexpected deaths.^3 8^ When such an event occurs, it should trigger the peer review process. This process is then followed by the implementation of corrective actions.^8^

It has long been known that audit filters perform poorly in this context.^9^ Replacing filters with trauma mortality prediction models has failed,^10^,^12^ likely because they were not developed to predict opportunities for improvement. No published research has evaluated prediction models for opportunities for improvement. We therefore aimed to develop models predicting opportunities for improvement in trauma care to screen patients for peer review and compare their performance to that of currently used audit filters.

## Methods

### Design

We conducted a registry-based study using all trauma patients included in both the Karolinska University Hospital trauma registry and the trauma care quality database between 2013 and 2022. The study was approved by the Swedish Ethical Review Authority (approval numbers 2021–02541 and 2021–03531).

### Study setting and population

The trauma centre at the Karolinska University Hospital in Solna, Sweden, manages approximately 1500 acute trauma patients each year.^13^

The Karolinska University Hospital trauma registry, which is part of the Swedish Trauma Registry,^13^ includes all patients admitted to the Karolinska University Hospital with trauma team activation, regardless of the injury severity score (ISS), as well as patients admitted without trauma team activation but found to have an ISS >9. The registry includes data on vital signs, times, injuries and interventions and demographics, according to the European consensus statement, the *Utstein Template for uniform reporting of data following major trauma*.^14^ The trauma care quality database includes data relevant to the peer review process, including audit filters, identified opportunities for improvement and proposed corrective actions.

The peer review process has evolved over time, but since 2017, a specialised nurse has reviewed the medical records of all trauma patients and flagged patients with potential opportunities for improvement using a set of audit filters (online supplemental table S1) and clinical experience. A second nurse performs a more indepth review of all flagged patients. Patients with suspected opportunities for improvement are then reviewed at a multidisciplinary conference, where the final decision on the presence of opportunities for improvement is made. All patients who die are reviewed at a separate conference that evaluates the preventability of the death and determines the presence of any opportunities for improvement. Before 2017, the process was less formalised, and a small group of clinicians involved in trauma care identified opportunities for improvement. We did not develop or implement audit filters as part of this study, but rather assessed if opportunities for improvement identified through the existing review process could be predicted using machine learning.

### Eligibility criteria

We included all patients screened for opportunities for improvement from the trauma registry and trauma care quality database between 1 January 2013 and 31 December 2022. Patients younger than 15 years of age were excluded because their clinical and review pathways differ from those of adults.

### Outcome

The models’ outcome is the presence of any opportunities for improvement, as determined by the peer review process. For descriptive purposes, these opportunities were categorised into clinical judgement errors, delays in treatment or diagnosis, missed diagnoses, technical errors, preventable deaths and other errors, based on frameworks from previous research and the established trauma quality literature.^10 15^

### Sample size considerations

The relationship between the number of predictors and the required sample size for different tabular models has not been well researched except for logistic regression.^16 17^ We used these guidelines to inform the number of predictors that we could include in our models, and we estimated that a sample size of 3452, which is equivalent to 80% of the available data from 2017 to 2020, would support 45 parameters, assuming a 6% event rate, an R^2^ of 0.11 and a target shrinkage of 0.9.

### Predictors

We selected predictors on the basis of current audit filters, standard demographics, previous research and expert opinions.^18^ The categorical predictors were gender, type of emergency procedure, highest level of care, reprioritisation, type of trauma alarm, discharge destination, death within 30 days, intubation status, completion of trauma CT and Glasgow Outcome Scale at discharge. The continuous predictors included age, vital signs on arrival, time to CT and intervention, ISS and length of stay. The final set of predictors comprised 18 variables with 49 corresponding parameters. Online supplemental table S3 shows all 18 predictors.

### Statistical analysis

The statistical analyses were conducted using R.^19^ We developed several prediction models using different learners, which are available in the supplementary material (online supplemental table S5), where we include the best-performing model: eXtreme Gradient Boosting (XGBoost)^20^ and the more interpretable model: logistic regression, here in the main text.

To evaluate the models, we used an add-1 year-in expanding window approach to best represent how the models would have performed if implemented prospectively. The years 2017–2022 were all used as separate validation hold-out sets in an iterative fashion. In each iteration, all the years prior to the current validation sample were used as training data. The training data were then split, and 80% of the data were used for training and 20% for calibration. We estimated 95% CIs for all performance metrics through a bootstrap of 1000 resamples for each validation sample.

#### Data preprocessing and imputation

We developed a preprocessor that rescaled continuous predictors using Yeo-Johnson’s power transformation^21^ and recorded categorical predictors as dummy variables via one-hot encoding. Missing predictors were imputed using k-nearest neighbors imputation. The k-nearest neighbors imputation was only fit on the training data. If blood pressure or respiratory rate data were missing but corresponding revised trauma score categorical values were available, we imputed the missing data using the mean of all patients in that category. To balance the training samples, we used the adaptive synthetic algorithm,^22^ which generates synthetic data, enabling us to upsample the opportunities for improvement outcomes at a balanced 1:1 ratio between outcome classes.

#### Model development

We developed logistic regression and XGBoost^20^ models using learners as implemented in the Tidymodels framework.^23^ All model hyperparameters were optimised on the training sample of each split using five-fold cross-validation through iterative Bayesian optimisation, encompassing all the parameters provided by the tidymodels framework.

#### Performance measurements

The performance of the prediction models and audit filters was assessed and compared in terms of calibration, discrimination, sensitivity and false positive rates for each validation sample. Calibration was measured using the integrated calibration index (ICI)^24^ and discrimination was measured using the area under the receiver operating characteristic (ROC) curve (AUC). The ICI was not calculated for the audit filters because they cannot estimate the probability of opportunities for improvement.

To determine the class probability cut-off for the two prediction models, we first configured them using Platt scaling on a 20% holdout sample from the training samples. We then determined the cut-off that produced a 95% sensitivity on this configuration sample and applied it to the holdout validation sample, called ‘high sensitivity configuration’. Additionally, we conducted an analysis to establish an ‘optimal’ cut-off threshold by identifying the point on the ROC curve that maximises the trade-off between sensitivity and specificity, called the ‘balanced configuration’.

#### Predictor importance

We calculated the predictor importance for the prediction models using permutation feature importance^25^ on the non-resampled validation samples. The importance of a feature was thus calculated by taking the average AUC performance when shuffling a feature’s data five times and comparing it to the model’s performance on non-shuffled data.

#### Code availability

The code used in this study is publicly available online: https://github.com/noacs-io/predicting-ofi-in-trauma under the MIT Licence.

### Patient and public involvement

Patients and/or the public were not involved in the design, conduct, reporting or dissemination plans of this research.

## Results

### Participants

**Figure 1:**
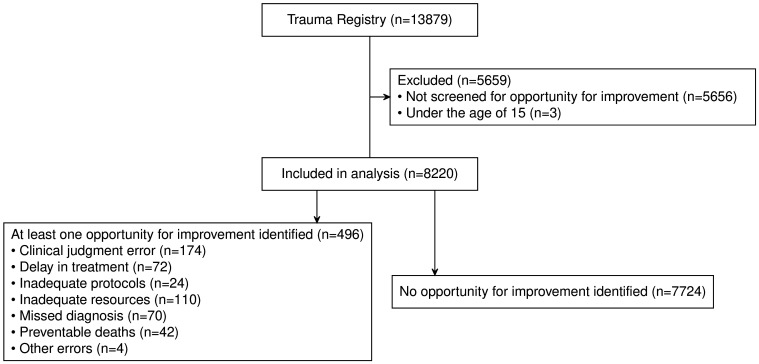
Flowchart describing the exclusions made and the distributions of opportunities for improvement categories. OFI, opportunity for improvement.

Among the 13 879 patients included in the registry between January 2013 and December 2022, 8220 (59%) patients were reviewed regarding the presence of opportunities for improvement, which were identified in 496 (6%) patients. The most common category of opportunities for improvement was clinical judgement errors (n=174, 35%) followed by inadequate resources (n=110, 22%). Among the 718 deaths, 42 (6%) were considered possibly preventable, accounting for 9% of all opportunities for improvement. Figure 1 details inclusions and exclusions as well as the frequency of each category of opportunities for improvement. Online supplemental table S2 shows the specific opportunities for improvement.

Patients with opportunities for improvement (mean=49 years, SD=21) were slightly older than patients without opportunities for improvement (mean=45 years, SD=21). The ISS was greater in patients with opportunities for improvement (mean=19, SD=11) than in patients without opportunities for improvement (mean=12, SD=13), and patients with opportunities for improvement had longer times (mean=271 min, SD=323) from hospital arrival to the first major intervention than patients without opportunities for improvement (mean=251 min, SD=351). Treatment frequencies also differed, with the greatest difference being in radiological interventions, where patients with opportunities for improvement (n=32, 6%) had more interventions than those without opportunities for improvement (n=69, 1%). Table 1 shows the characteristics of all reviewed patients.

**Table 1:** Demographic and clinical characteristics of patients screened for opportunities for improvement

	Opportunity for improvement (n=496)	No opportunity for improvement (n=7724)	Overall (n=8220)
Age (years)			
Mean (SD)	49 (22)	45 (21)	45 (21)
Median (Q1, Q3)	49 (30, 67)	43 (27, 61)	43 (27, 61)
Sex			
Female	136 (27%)	2388 (31%)	2524 (31%)
Male	360 (73%)	5336 (69%)	5696 (69%)
Dead at 30 days			
Yes	41 (8%)	677 (9%)	718 (9%)
No	453 (91%)	7038 (91%)	7491 (91%)
Missing	2 (<1%)	9 (<1%)	11 (<1%)
Highest level of care			
Emergency department	22 (4%)	1467 (19%)	1489 (18%)
General ward	116 (23%)	2920 (38%)	3036 (37%)
Operation theatre	141 (28%)	1438 (19%)	1579 (19%)
Specialist ward/intermediate ward	50 (10%)	336 (4%)	386 (5%)
Intensive care unit	167 (34%)	1563 (20%)	1730 (21%)
Injury severity score			
Mean (SD)	19 (11)	12 (13)	12 (13)
Median (Q1, Q3)	17 (10, 25)	9 (1, 17)	9 (2, 17)
Missing	0 (0%)	10 (<1%)	10 (<1%)
ED Respiratory rate			
Mean (SD)	19 (5)	18 (5)	18 (5)
Median (Q1, Q3)	18 (16, 20)	18 (16, 20)	18 (16, 20)
Missing	51 (10%)	812 (11%)	863 (10%)
ED Glasgow Coma Scale			
Mean (SD)	14 (3)	14 (2)	14 (2)
Median (Q1, Q3)	15 (14, 15)	15 (14, 15)	15 (14, 15)
Missing	49 (10%)	811 (11%)	860 (10%)
ED Systolic blood pressure (mm Hg)			
Mean (SD)	133 (34)	133 (33)	133 (33)
Median (Q1, Q3)	135 (118, 150)	135 (120, 150)	135 (120, 150)
Missing	0 (0%)	13 (<1%)	13 (<1%)
Time to first CT (minutes)			
Mean (SD)	76 (129)	70 (134)	70 (134)
Median (Q1, Q3)	40 (25, 72)	33 (21, 66)	33 (22, 67)
Missing	42 (8%)	949 (12%)	991 (12%)
Time to first major intervention (minutes)			
Mean (SD)	271 (323)	251 (351)	253 (348)
Median (Q1, Q3)	143 (91, 284)	102 (50, 251)	106 (54, 260)
Missing	230 (46%)	5673 (73%)	5903 (72%)
Emergency procedure			
Thoracotomy	8 (2%)	97 (1%)	105 (1%)
Laparotomy	28 (6%)	213 (3%)	241 (3%)
Pelvic packing	0 (0%)	5 (<1%)	5 (<1%)
Revascularisation	12 (2%)	37 (<1%)	49 (1%)
Radiological intervention	32 (6%)	69 (1%)	101 (1%)
Craniotomy	42 (8%)	240 (3%)	282 (3%)
Intracranial pressure measurement	13 (3%)	90 (1%)	103 (1%)
Other	131 (26%)	1305 (17%)	1436 (17%)
No procedure	230 (46%)	5666 (73%)	5896 (72%)
Missing	0 (0%)	2 (<1%)	2 (<1%)

The vital signs are the first measured vital signs in the emergency department. Time to first Computed Tomography (CT) and Time to time to first major intervention: measured in minutes from arrival at the hospital.

CT, Computed Tomography; ED, emergency department.

### Model development, specification and performance

The frequency of opportunities for improvement varied between 2017 and 2022, with the highest occurring in 2017 (n=112, 9%) and the lowest occurring in 2018 (n=36, 3%). The annual characteristics are provided in online supplemental table S4.

**Figure 2:**
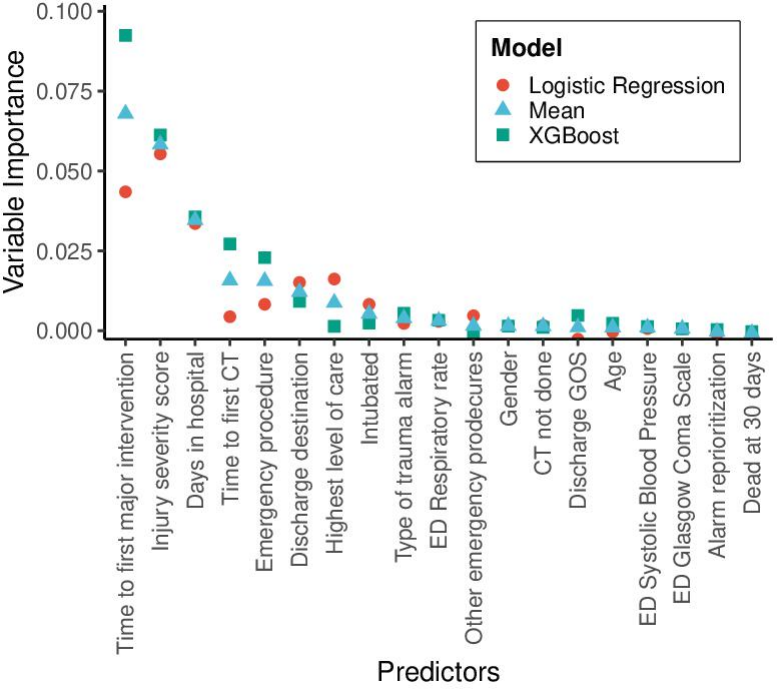
Average permuted variable importance for each predictor between 2017 and 2022. Each point indicates the average performance degradation in terms of the AUC of the corresponding model after randomly shuffling the data for that predictor, showing its contribution to the model’s performance. AUC, area under the receiver operating characteristic curve; ED, emergency department; GOS, Glasgow Outcome Scale.

The most important predictor was time to first major intervention, followed by the ISS. Figure 2 shows the average predictor importance for all years between 2017 and 2022 for all the predictors.

When pooling the results from each year (table 2), the audit filters had an AUC of 0.62 (0.61, 0.64), a sensitivity of 0.91 (0.89, 0.94) and a false positive rate of 0.67 (0.66, 0.68). In the high sensitivity configuration, the XGBoost model achieved a sensitivity of 0.90 (0.87, 0.93) with a significantly lower false positive rate of 0.63 (0.61, 0.64) than the audit filters did. The logistic regression model achieved a sensitivity of 0.94 (0.91, 0.96) and a false positive rate of 0.72 (0.71, 0.73). Both models demonstrated good calibration, with ICIs of 0.02 (0.01, 0.02) for XGBoost and 0.02 (0.02, 0.03) for logistic regression. In the balanced configuration, the XGBoost model had a sensitivity of 0.58 (0.53, 0.63) and a false positive rate of 0.25 (0.24, 0.26). The logistic regression model yielded a sensitivity of 0.56 (0.51, 0.61) and a false positive rate of 0.27 (0.26, 0.28).

**Table 2:** Pooled performance metrics for predicting opportunities for improvement across the years 2017–2022.

Model	AUC	False positive rate	Sensitivity	ICI
Audit filters				
Audit filters	0.62 (0.61, 0.64)	0.67 (0.66, 0.68)	0.91 (0.89, 0.94)	–
High sensitivity calibration				
Logistic regression	0.71 (0.69, 0.74)	0.72 (0.71, 0.73)	0.94 (0.91, 0.96)	0.02 (0.02, 0.03)
XGBoost	0.74 (0.72, 0.77)	0.63 (0.61, 0.64)	0.90 (0.87, 0.93)	0.02 (0.01, 0.02)
Performance differences between audit filters and high sensitivity calibration				
Logistic regression	0.09 (0.07, 0.12)	0.05 (0.04, 0.07)	0.03 (-0.00, 0.05)	–
XGBoost	0.12 (0.10, 0.14)	−0.05 (-0.06, –0.03)	−0.01 (-0.05, 0.02)	–
Optimal calibration				
Logistic regression	0.71 (0.69, 0.74)	0.27 (0.26, 0.28)	0.56 (0.51, 0.61)	0.02 (0.02, 0.03)
XGBoost	0.74 (0.72, 0.77)	0.25 (0.24, 0.26)	0.58 (0.53, 0.63)	0.02 (0.01, 0.02)
Performance differences between audit filters and optimal calibration				
Logistic regression	0.09 (0.07, 0.12)	−0.40 (-0.42, –0.39)	−0.35 (-0.40,–0.30)	–
XGBoost	0.12 (0.10, 0.14)	−0.42 (-0.44,–0.41)	−0.34 (-0.39,–0.28)	–

Pooled performance measures for the audit filters, logistic regression and XGBoost model using the expanding window add-1 year-in approach. The performance differences are calculated by subtracting the audit filter performance values from the corresponding model value. ICI is not calculated for audit filters since they do not output prediction probabilities.

AUC, area under the receiver operating characteristic curve; ICI, integrated calibration index.

When comparing the XGBoost model to the logistic regression model (online supplemental table S6), an overall AUC improvement of 0.03 (95% CI: 0.01 to 0.05) in favour of XGBoost was observed. In the high sensitivity configuration, the XGBoost model demonstrated a slightly lower sensitivity (−0.04; 95% CI: −0.07 to –0.01) but achieved a substantially reduced false positive rate (−0.10; 95% CI: −0.11 to –0.09) compared with logistic regression. However, in the optimal calibration configuration, no statistically significant differences were observed between the two models.

**Figure 3:**
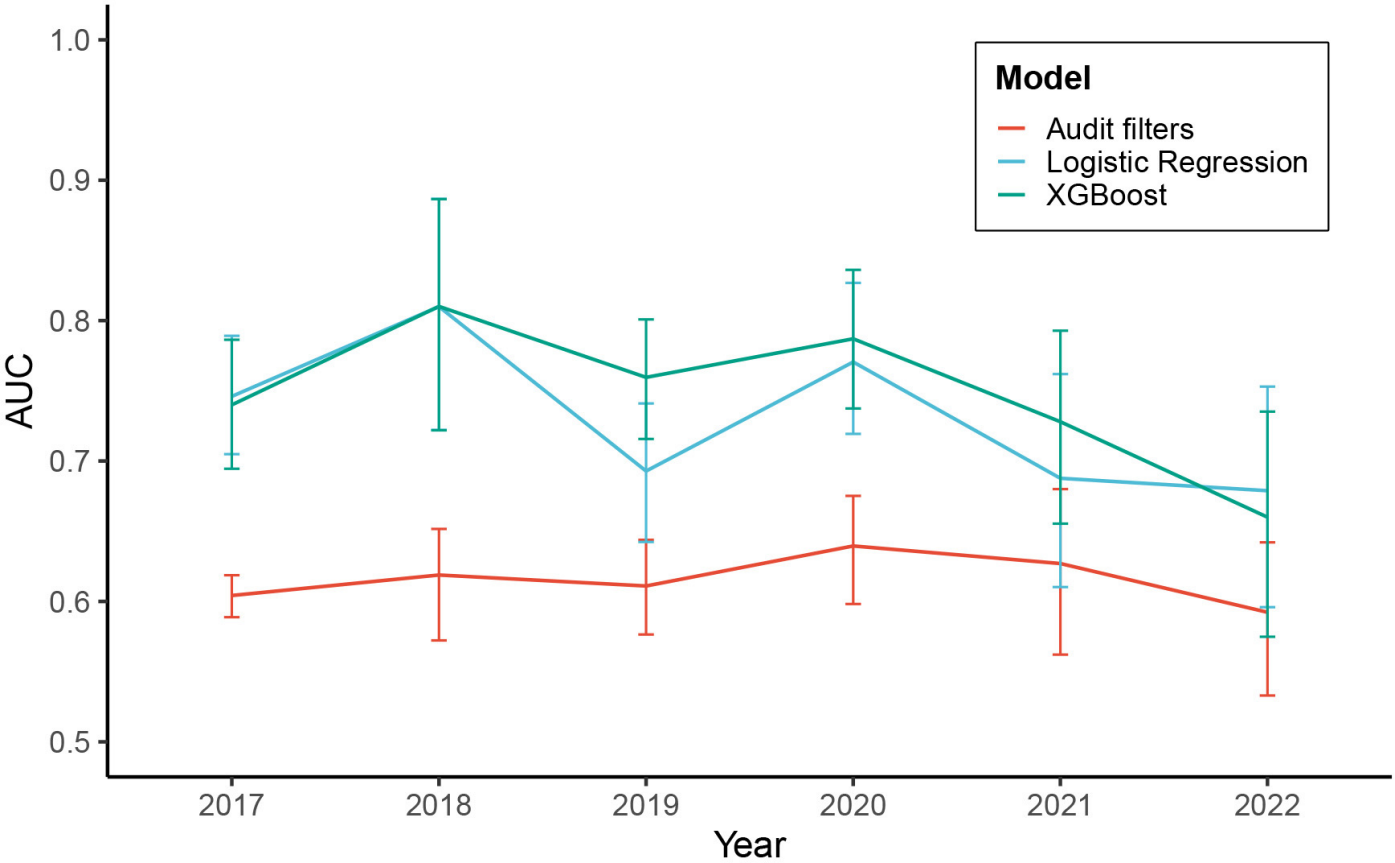
AUC performance in predicting opportunities for improvement by model and year. The error bars indicate the 95% CI. AUC, area under the receiver operating characteristic curve

Figure 3 shows the annual AUC values for each year between 2017 and 2022. The annual sample size is displayed in online supplemental table S4. Online supplemental figures S1–S3 show the annual sensitivity, false positive rate and ROC curves between 2017 and 2022. Online supplemental table S5 displays metrics for more tested models, as well as the absolute true positive and false positive rates for 2017–2022.

## Discussion

The XGBoost model outperformed audit filters in predicting opportunities for improvement among adult trauma patients. The logistic regression model displayed a higher sensitivity at the cost of a higher false positive rate. The performances of both models were modest, and the audit filters exhibited poor performance. Unlike audit filters, these models can be configured towards specific goals where we test two configuration strategies: one prioritising a higher sensitivity with a moderate reduction in false positive rate and another accepting a moderate loss in sensitivity for a substantial reduction in false positive rate. This adaptability allows these models to better balance identifying opportunities for improvement and managing the screening burden, which in combination with a superior overall performance offers potential advantages over traditional audit filters. Additionally, unlike audit filters’ binary output, the probability scores from these models enable identification of ‘high-risk’ cases along a continuous spectrum, allowing for more nuanced prioritisation of reviews.

### Interpretation and generalisability

While the use of audit filters when screening for opportunities for improvement remains the current state-of-the-art technique for trauma quality improvement programmes, their effectiveness, especially in the mature trauma system, has long been questioned.^8 9^ The static nature of audit filters makes them less effective as the trauma system adapts, potentially requiring frequent changes over time. The adaptive nature of machine learning models offers a promising solution, allowing the models and subsequently selected patient cases to change as the models develop over time. While our study suggests this as a possibility, prospective implementation is needed for true evaluation.

The XGBoost model in the high sensitivity configuration had a similar sensitivity to that of the audit filters but achieved a 7% (n=46) reduction in the annual screening burden. This reduction in false positives is further highlighted when configuring towards balanced performance, reducing the screening burden by 63% (n=426) while identifying 37% (n=19) fewer opportunities for improvement annually. Although the reduction in sensitivity is not ideal, this trade-off should be considered given that trauma systems may forgo peer review altogether owing to the high false positive rate of audit filters. Thus, the significant reduction in false positive rate could offer benefits for settings with limited resources.

Additionally, the need for extra human review as a consequence of the high false positive rate before the mortality and morbidity review risks introducing bias, reducing the intended multidisciplinary approach. Mortality prediction models have been suggested as a solution; however, they perform poorly and are only applicable in mortality-related cases.^10^ The balanced models offer a potential solution where over 80% of the flagged cases contain opportunities for improvement. Cases can therefore be brought directly from standard trauma registries to the mortality and morbidity review without additional human prescreening. The possibility to configure these models therefore represents a tool for high-yield selection in contexts that want to include morbidity cases while protecting the intended multidisciplinary approach of the final review.

A systematic review and meta-analysis by Zhang *et al* investigated the performance of different machine learning applications and learners in the trauma setting and found that similar performances were often found using logistic regression compared with more complex machine learning models.^26^ Our study showed that XGBoost had a small, but significant, performance advantage compared with logistic regression; however, both performed modestly. Instead, a substantial performance increase would probably require both higher quantity and quality of data, for example, higher-resolution data such as vital sign series, linkage to other national registries or text analysis of electronic health records using large language models, alongside defined, complete and consistent opportunities for improvement classifications. However, in the current analysis, we opted to exclude additional data sources to maintain external validity and general feasibility, ensuring our models remain easily applicable in settings with standard registries following the *Utstein Template for uniform reporting of data following major trauma*.^14^

### Limitations

Importantly, our models’ perfomances are most likely underestimated due to two limitations. First, the addition of a year-by-year approach to simulate prospective implementation resulted in small sample sizes between 2017 and 2020, leading to poorer configuration. Second, these models are only evaluated against opportunities for improvement identified within the current peer review system. Compared with previous studies, the low frequency of opportunities for improvement in this study suggests potential false negatives.^10 27 28^ These false negatives favour the models and increase their performance, as they were missed by the current audit filter and peer review system.

While defined as a binary variable, opportunities for improvement include a diverse set of outcomes ranging from preventable deaths to a lack of communication. The heterogeneity of these outcomes represents a range of clinical events, each of which is likely correlated with different predictors. In addition, machine learning models struggle to handle rare events, and despite being an aggregate of all previously identified errors, the opportunity for improvement frequency is only 6%; as a result, opportunities for improvement are a considerable predictive challenge. Furthermore, there lacks standardisation of audit filters between institutions. The locally-developed audit filters used may differ from those used elsewhere, which affects the generalisability of our findings to other trauma centres.

Another potential risk is a ‘data shift’. Owing to feasibility, mortality and morbidity reviews and corresponding corrective actions can focus only on a subset of opportunities for improvement at any given time. Hence, correctly flagged opportunities for improvement might not be registered since the system must prioritise other areas in need of correction. If human resources could be removed from basic screening tasks by reducing false positives, they could be allocated towards more indepth reviews, reducing the need to prioritise opportunities for improvement subgroups.

## Conclusions

Importantly, perfect performance is far from expected. Comparing these models with entire systems via a combination of quantitative screening and several human reviews, including a multidisciplinary review, is unfair and not the goal of this paper. Instead, we strive to facilitate quality improvement efforts through a combination of human and artificial intelligence. Our models are designed to identify cases for human review, as the currently used audit filters. Compared with audit filters, these models offer increased overall performance and the option to balance and optimise the trade-off between screening burden and sensitivity goals, giving each trauma quality improvement programme the potential to standardise and automate a part of the review system, in a way that complements human efforts.

## Supplementary material

10.1136/bmjopen-2025-099624 online supplemental file 1

## Data Availability

No data are available.
