# Supplementary material for: Predicting opportunities for improvement in trauma care using machine learning: a retrospective registry-based study at a major trauma centre
Source: BMJ Open. 2025 Jun 6;15(6):e099624. doi: 10.1136/bmjopen-2025-099624 (PMC12161345; doi:10.1136/bmjopen-2025-099624)
Supplement: online supplemental file 1 [file bmjopen-15-6-s001.pdf]

# Supplementary material

## Predicting Opportunities for Improvement in Trauma Care Using Machine Learning: A Retrospective Registry-Based Study at a Major Trauma Centre

Jonatan Attergrim Kelvin Szolnoky Lovisa Strömmer Olof Brattström
Gunilla Wihlke Martin Jacobsson Martin Gerdin Wärnberg

Table S1: Current audit filters at Karolinska University Hospital in Stockholm, Sweden, to detect opportunities for improvement in trauma care.

| **A specialized nurse checks if any of the following audit criteria are met:** |
|---|
| ✓ In hospital death or death within 30-days |
| ✓ Systolic blood pressure less than 90 mmHg |
| ✓ Glasgow coma scale less than 9 and not intubated |
| ✓ Injury severity score greater than 15, but the patient was not admitted to the intensive care unit |
| ✓ Injury severity score greater than 15, but no trauma team activation |
| ✓ Time to acute intervention more than 60 minutes from arrival at the hospital |
| ✓ Time to computed tomography more than 30 minutes from arrival at the hospital |
| ✓ No anticoagulant therapy administered within 72 hours after a traumatic brain injury |
| ✓ Presence of cardiopulmonary resuscitation with thoracotomy |
| ✓ Presence of a liver or spleen injury |
| ✓ Massive transfusion, defined as 10 or more units of packed red blood cells within 24 hours |

Table S2: Number of opportunitites for improvement per category and subcategory (n = 496)

| Category (n) | Subcategory (n) |
|---|---|
| **Clinical judgment error (174)**<br>An incorrect management strategy, despite sufficient information and resources, leading to patient harm. | • Triage in the ED: 64<br>• Level of care: 48<br>• Patient management: 46<br>• Communication: 16 |
| **Delay in treatment (72)**<br>Inappropriate delays from arrival to treatment or imaging leading to harm. | • Delay to surgery: 47<br>• Delay to CT: 25 |
| **Inadequate protocols (24)**<br>Lacking or not following protocols/guidelines resulting in patient harm. | • Trauma criteria/guidelines: 22<br>• Inadequate routine: 2 |
| **Inadequate resources (110)**<br>Lack of resources, including personnel, or material, leading to patient harm. | • Competence: 1<br>• Resources: 73<br>• Logistics/technical: 36 |
| **Missed diagnosis (70)**<br>Failure to appropriately diagnose an injury or condition despite having sufficient information and resources. | • Missed injury: 69<br>• Tertiary survey: 1 |
| **Preventable death (42)**<br>Mortality that could have been prevented in the optimal trauma care system under optimal treatment conditions as decided by the MoM-review. | • Preventable death: 4<br>• Possible preventable death: 38 |
| **Other errors (4)** | • Other: 1<br>• Patient management/logistics: 1<br>• Prehospital management: 1<br>• Neurosurgery: 1 |

Definitions of abbreviations: MoM = Mortality and morbidity; ED = Emergency Department; CT = Computed tomography.

Table S3: Demographic and clinical characteristics of patients screened for opportunities for improvement, for all predictors used in the machine learning models.

| | Opportunity for improvement | No opportunity for improvement | Overall |
|---|---|---|---|
| | (N=496) | (N=7724) | (N=8220) |
| **Age (years)** | | | |
| Mean (SD) | 49 (21) | 45 (21) | 45 (21) |
| Median (Q1, Q3) | 49 (30, 67) | 43 (27, 61) | 43 (27, 61) |
| **Sex** | | | |
| Female | 136 (27%) | 2388 (31%) | 2524 (31%) |
| Male | 360 (73%) | 5336 (69%) | 5696 (69%) |
| **Injury severity score** | | | |
| Mean (SD) | 19 (11) | 12 (13) | 12 (13) |
| Median (Q1, Q3) | 17 (10, 25) | 9 (1, 17) | 9 (2, 17) |
| Missing | 0 (0%) | 10 (<1%) | 10 (<1%) |
| **ED Respiratory rate** | | | |
| Mean (SD) | 19 (5) | 18 (5) | 18 (5) |
| Median (Q1, Q3) | 18 (16, 20) | 18 (16, 20) | 18 (16, 20) |
| Missing | 51 (10%) | 812 (11%) | 863 (10%) |
| **ED Glasgow Coma Scale** | | | |
| Mean (SD) | 14 (3) | 14 (2) | 14 (2) |
| Median (Q1, Q3) | 15 (14, 15) | 15 (14, 15) | 15 (14, 15) |
| Missing | 49 (10%) | 811 (11%) | 860 (10%) |
| **ED Systolic Blood Pressure (mmHg)** | | | |
| Mean (SD) | 133 (34) | 133 (33) | 133 (33) |
| Median (Q1, Q3) | 135 (118, 150) | 135 (120, 150) | 135 (120, 150) |
| Missing | 0 (0%) | 13 (<1%) | 13 (<1%) |
| **Emergency procedure** | | | |
| Thoracotomy | 8 (2%) | 97 (1%) | 105 (1%) |
| Laparotomy | 28 (6%) | 213 (3%) | 241 (3%) |
| Pelvis Packing | 0 (0%) | 5 (<1%) | 5 (<1%) |
| Revascularization | 12 (2%) | 37 (<1%) | 49 (1%) |
| Radiological intervention | 32 (6%) | 69 (1%) | 101 (1%) |
| Craniotomy | 42 (8%) | 240 (3%) | 282 (3%) |
| Intracranial pressure measurement | 13 (3%) | 90 (1%) | 103 (1%) |
| Other | 131 (26%) | 1305 (17%) | 1436 (17%) |
| No procedure | 230 (46%) | 5666 (73%) | 5896 (72%) |
| Missing | 0 (0%) | 2 (<1%) | 2 (<1%) |
| **Other emergency prodecures** | | | |
| Chest drain | 40 (8%) | 399 (5%) | 439 (5%) |
| External fixation of fracture | 22 (4%) | 163 (2%) | 185 (2%) |
| Major fracture surgery | 37 (7%) | 300 (4%) | 337 (4%) |
| Wound revision in OR | 27 (5%) | 387 (5%) | 414 (5%) |
| Other action | 5 (1%) | 56 (1%) | 61 (1%) |
| No procedure | 230 (46%) | 5666 (73%) | 5896 (72%) |
| Missing | 135 (27%) | 753 (10%) | 888 (11%) |
| **Time to first major intervention (minutes)** | | | |
| Mean (SD) | 271 (323) | 251 (351) | 253 (348) |
| Median (Q1, Q3) | 143 (91, 284) | 102 (50, 251) | 106 (54, 260) |
| Missing | 230 (46%) | 5673 (73%) | 5903 (72%) |
| **Type of trauma alarm** | | | |
| Trauma alarm level 1 | 196 (40%) | 4068 (53%) | 4264 (52%) |
| Trauma alarm level 2 | 81 (16%) | 1890 (24%) | 1971 (24%) |
| No trauma alarm | 53 (11%) | 544 (7%) | 597 (7%) |
| Missing | 166 (33%) | 1222 (16%) | 1388 (17%) |
| **Alarm reprioritization** | | | |
| No reprioritization | 313 (63%) | 6300 (82%) | 6613 (80%) |
| Reprioritization to level 1 | 15 (3%) | 150 (2%) | 165 (2%) |
| Reprioritization to level 2 | 2 (<1%) | 43 (1%) | 45 (1%) |
| Alarm cancelled | 0 (0%) | 9 (<1%) | 9 (<1%) |
| Missing | 166 (33%) | 1222 (16%) | 1388 (17%) |
| **CT not done** | | | |

Table S3: Demographic and clinical characteristics of patients screened for opportunities for improvement, for all predictors used in the machine learning models. (cont.)

| | Opportunity for improvement | No opportunity for improvement | Overall |
|---|---|---|---|
| CT done | 454 (92%) | 6779 (88%) | 7233 (88%) |
| CT not done | 42 (8%) | 945 (12%) | 987 (12%) |
| **Time to first CT (minutes)** | | | |
| Mean (SD) | 76 (129) | 70 (134) | 70 (134) |
| Median (Q1, Q3) | 40 (25, 72) | 33 (21, 66) | 33 (22, 67) |
| Missing | 42 (8%) | 949 (12%) | 991 (12%) |
| **Intubated** | | | |
| Inhospital | 80 (16%) | 609 (8%) | 689 (8%) |
| Not intubated | 385 (78%) | 6548 (85%) | 6933 (84%) |
| Prehospital | 31 (6%) | 566 (7%) | 597 (7%) |
| Missing | 0 (0%) | 1 (<1%) | 1 (<1%) |
| **Highest level of care** | | | |
| Emergency department | 22 (4%) | 1467 (19%) | 1489 (18%) |
| General ward | 116 (23%) | 2920 (38%) | 3036 (37%) |
| Operation Theatre | 141 (28%) | 1438 (19%) | 1579 (19%) |
| Specialist ward/Intermediate ward | 50 (10%) | 336 (4%) | 386 (5%) |
| Intensive care unit | 167 (34%) | 1563 (20%) | 1730 (21%) |
| **Discharge destination** | | | |
| Home | 199 (40%) | 4847 (63%) | 5046 (61%) |
| Rehab | 199 (40%) | 1380 (18%) | 1579 (19%) |
| Morgue | 35 (7%) | 611 (8%) | 646 (8%) |
| ICU (higher care level) | 2 (<1%) | 41 (1%) | 43 (1%) |
| ICU (same care level) | 10 (2%) | 112 (1%) | 122 (1%) |
| Other department | 38 (8%) | 435 (6%) | 473 (6%) |
| Psychiatric care | 13 (3%) | 296 (4%) | 309 (4%) |
| Missing | 0 (0%) | 2 (<1%) | 2 (<1%) |
| **Days in hospital** | | | |
| Mean (SD) | 12 (14) | 6 (14) | 7 (14) |
| Median (Q1, Q3) | 7 (3, 13) | 3 (2, 6) | 3 (2, 7) |
| **Dead at 30 days** | | | |
| Yes | 41 (8%) | 677 (9%) | 718 (9%) |
| No | 453 (91%) | 7038 (91%) | 7491 (91%) |
| Missing | 2 (<1%) | 9 (<1%) | 11 (<1%) |
| **Discharge glascow outcome scale** | | | |
| Mean (SD) | 3 (1) | 4 (1) | 4 (1) |
| Median (Q1, Q3) | 3 (3, 4) | 4 (3, 5) | 4 (3, 5) |
| Missing | 0 (0%) | 1 (<1%) | 1 (<1%) |

Definition of abbreviations: OFI = Opportunity for Improvement; ED = Emergency Department.
Time to first CT and Time to first major intervention: Measured in minutes from arrival at the hospital

Table S4: Demographic and clinical characteristics of patients screened for opportunities for improvement, by year.

| | 2013-2016 | 2017 | 2018 | 2019 | 2020 | 2021 | 2022 | Overall |
|---|---|---|---|---|---|---|---|---|
| | (N=761) | (N=1282) | (N=1320) | (N=1181) | (N=1324) | (N=1265) | (N=1087) | (N=8220) |
| **Opportunity for improvement** | | | | | | | | |
| Opportunity for improvement | 95 (12%) | 112 (9%) | 36 (3%) | 99 (8%) | 71 (5%) | 37 (3%) | 46 (4%) | 496 (6%) |
| No opportunity for improvement | 666 (88%) | 1170 (91%) | 1284 (97%) | 1082 (92%) | 1253 (95%) | 1228 (97%) | 1041 (96%) | 7724 (94%) |
| **Age (years)** | | | | | | | | |
| Mean (SD) | 50 (23) | 45 (21) | 44 (20) | 45 (21) | 44 (21) | 45 (21) | 47 (22) | 45 (21) |
| Median (Q1, Q3) | 48 (28, 69) | 42 (27, 59) | 41 (27, 59) | 43 (26, 61) | 41 (26, 59) | 43 (27, 61) | 45 (28, 64) | 43 (27, 61) |
| **Sex** | | | | | | | | |
| Female | 225 (30%) | 408 (32%) | 418 (32%) | 348 (29%) | 384 (29%) | 389 (31%) | 352 (32%) | 2524 (31%) |
| Male | 536 (70%) | 874 (68%) | 902 (68%) | 833 (71%) | 940 (71%) | 876 (69%) | 735 (68%) | 5696 (69%) |
| **Dead at 30 days** | | | | | | | | |
| Yes | 232 (30%) | 92 (7%) | 73 (6%) | 88 (7%) | 83 (6%) | 74 (6%) | 76 (7%) | 718 (9%) |
| No | 527 (69%) | 1184 (92%) | 1247 (94%) | 1092 (92%) | 1241 (94%) | 1189 (94%) | 1011 (93%) | 7491 (91%) |
| Missing | 2 (<1%) | 6 (<1%) | 0 (0%) | 1 (<1%) | 0 (0%) | 2 (<1%) | 0 (0%) | 11 (<1%) |
| **Highest level of care** | | | | | | | | |
| Emergency department | 37 (5%) | 316 (25%) | 357 (27%) | 232 (20%) | 212 (16%) | 176 (14%) | 159 (15%) | 1489 (18%) |
| General ward | 185 (24%) | 467 (36%) | 469 (36%) | 413 (35%) | 528 (40%) | 567 (45%) | 407 (37%) | 3036 (37%) |
| Operation Theatre | 196 (26%) | 227 (18%) | 237 (18%) | 222 (19%) | 263 (20%) | 218 (17%) | 216 (20%) | 1579 (19%) |
| Specialist ward/Intermediate ward | 23 (3%) | 21 (2%) | 37 (3%) | 61 (5%) | 78 (6%) | 82 (6%) | 84 (8%) | 386 (5%) |
| Intensive care unit | 320 (42%) | 251 (20%) | 220 (17%) | 253 (21%) | 243 (18%) | 222 (18%) | 221 (20%) | 1730 (21%) |
| **Injury severity score** | | | | | | | | |
| Mean (SD) | 24 (17) | 11 (12) | 10 (12) | 11 (12) | 11 (12) | 11 (12) | 12 (12) | 12 (13) |
| Median (Q1, Q3) | 21 (14, 29) | 9 (1, 16) | 5 (1, 14) | 9 (1, 14) | 9 (1, 16) | 9 (1, 17) | 9 (2, 17) | 9 (2, 17) |
| Missing | 0 (0%) | 3 (<1%) | 0 (0%) | 1 (<1%) | 3 (<1%) | 1 (<1%) | 2 (<1%) | 10 (<1%) |
| **Time to first CT (minutes)** | | | | | | | | |
| Mean (SD) | 71 (123) | 86 (129) | 71 (119) | 66 (126) | 77 (172) | 58 (131) | 61 (123) | 70 (134) |
| Median (Q1, Q3) | 35 (25, 60) | 49 (30, 96) | 37 (24, 77) | 32 (20, 63) | 27 (18, 60) | 27 (19, 48) | 28 (20, 55) | 33 (22, 67) |
| Missing | 125 (16%) | 143 (11%) | 142 (11%) | 138 (12%) | 163 (12%) | 141 (11%) | 139 (13%) | 991 (12%) |
| **Time to first major intervention (minutes)** | | | | | | | | |
| Mean (SD) | 214 (307) | 213 (306) | 236 (321) | 203 (297) | 283 (382) | 323 (413) | 299 (372) | 253 (348) |
| Median (Q1, Q3) | 108 (22, 200) | 100 (37, 198) | 110 (62, 236) | 99 (50, 193) | 105 (55, 305) | 116 (63, 412) | 110 (60, 378) | 106 (54, 260) |
| Missing | 334 (44%) | 1002 (78%) | 1028 (78%) | 865 (73%) | 973 (73%) | 929 (73%) | 772 (71%) | 5903 (72%) |

Time to first CT and Time to first major intervention: Measured in minutes from arrival at the hospital.

Table S5: Complete pooled performance metrics for all machine learning models and for years 2017-2022.

| **Model** | **AUC** | **False positive rate** | **Sensitivity** | **True positives** | **False positives** | **ICI** |
|---|---|---|---|---|---|---|
| | | | **Audit filters** | | | |
| Audit filters | 0.62 (0.61, 0.64) | 0.67 (0.66, 0.68) | 0.91 (0.89, 0.94) | 365.68 (329.00, 404.02) | 4734.73 (4655.00, 4818.02) | - |
| | | | **High sensitivity calibration** | | | |
| CatBoost | 0.72 (0.69, 0.74) | 0.78 (0.77, 0.79) | 0.94 (0.92, 0.96) | 377.78 (342.00, 416.02) | 5496.32 (5423.00, 5573.02) | 0.02 (0.02, 0.02) |
| Decision tree | 0.70 (0.67, 0.73) | 0.67 (0.66, 0.68) | 0.91 (0.89, 0.94) | 365.89 (328.00, 404.00) | 4735.82 (4653.95, 4816.02) | 0.02 (0.01, 0.02) |
| LightGBM | 0.74 (0.71, 0.76) | 0.65 (0.64, 0.66) | 0.92 (0.89, 0.94) | 366.92 (331.00, 406.00) | 4588.06 (4506.97, 4668.00) | 0.02 (0.01, 0.02) |
| Logistic Regression | 0.71 (0.69, 0.74) | 0.72 (0.71, 0.73) | 0.94 (0.91, 0.96) | 375.94 (339.00, 414.02) | 5105.33 (5026.98, 5183.02) | 0.02 (0.02, 0.03) |
| Random Forest | 0.74 (0.72, 0.77) | 0.69 (0.67, 0.70) | 0.93 (0.90, 0.95) | 370.62 (334.97, 410.00) | 4841.10 (4763.98, 4924.03) | 0.02 (0.01, 0.02) |
| XGBoost | 0.74 (0.72, 0.77) | 0.63 (0.61, 0.64) | 0.90 (0.87, 0.93) | 359.96 (324.98, 398.00) | 4415.80 (4334.00, 4500.00) | 0.02 (0.01, 0.02) |
| | | | **Performance differences between audit filters and high sensitivity calibration** | | | |
| CatBoost | 0.10 (0.07, 0.12) | 0.11 (0.09, 0.12) | 0.03 (-0.00, 0.06) | 12.09 (-0.00, 24.00) | 761.59 (663.98, 861.03) | - |
| Decision tree | 0.08 (0.05, 0.11) | 0.00 (-0.01, 0.01) | 0.00 (-0.04, 0.04) | 0.20 (-15.00, 15.00) | 1.09 (-100.02, 105.05) | - |
| LightGBM | 0.11 (0.09, 0.14) | -0.02 (-0.03, -0.01) | 0.00 (-0.03, 0.04) | 1.24 (-12.00, 14.00) | -146.68 (-241.00, -47.95) | - |
| Logistic Regression | 0.09 (0.07, 0.12) | 0.05 (0.04, 0.07) | 0.03 (-0.00, 0.05) | 10.25 (-2.00, 22.00) | 370.60 (264.98, 467.02) | - |
| Random Forest | 0.12 (0.10, 0.15) | 0.02 (0.00, 0.03) | 0.01 (-0.02, 0.05) | 4.93 (-9.00, 19.00) | 106.37 (9.00, 205.02) | - |
| XGBoost | 0.12 (0.10, 0.14) | -0.05 (-0.06, -0.03) | -0.01 (-0.05, 0.02) | -5.72 (-18.02, 8.00) | -318.93 (-414.00, -218.98) | - |
| | | | **Optimal calibration** | | | |
| CatBoost | 0.72 (0.69, 0.74) | 0.27 (0.26, 0.28) | 0.56 (0.52, 0.61) | 225.94 (196.97, 256.00) | 1896.02 (1820.00, 1968.00) | 0.02 (0.02, 0.02) |
| Decision tree | 0.70 (0.67, 0.73) | 0.31 (0.29, 0.32) | 0.56 (0.51, 0.61) | 224.29 (196.00, 255.00) | 2156.24 (2076.97, 2233.00) | 0.02 (0.01, 0.02) |
| LightGBM | 0.74 (0.71, 0.76) | 0.26 (0.25, 0.27) | 0.59 (0.54, 0.64) | 237.12 (206.98, 268.02) | 1805.03 (1735.97, 1875.00) | 0.02 (0.01, 0.02) |
| Logistic Regression | 0.71 (0.69, 0.74) | 0.27 (0.26, 0.28) | 0.56 (0.51, 0.61) | 223.98 (194.00, 254.00) | 1895.82 (1824.00, 1970.00) | 0.02 (0.02, 0.03) |
| Random Forest | 0.74 (0.72, 0.77) | 0.26 (0.25, 0.27) | 0.60 (0.55, 0.64) | 238.61 (209.00, 270.02) | 1803.23 (1735.00, 1872.00) | 0.02 (0.01, 0.02) |
| XGBoost | 0.74 (0.72, 0.77) | 0.25 (0.24, 0.26) | 0.58 (0.53, 0.63) | 230.73 (201.00, 261.00) | 1755.80 (1687.98, 1832.00) | 0.02 (0.01, 0.02) |
| | | | **Performance differences between audit filters and optimal calibration** | | | |
| CatBoost | 0.10 (0.07, 0.12) | -0.40 (-0.42, -0.39) | -0.35 (-0.40, -0.30) | -139.74 (-165.00, -114.98) | -2838.71 (-2935.03, -2741.00) | - |
| Decision tree | 0.08 (0.05, 0.11) | -0.37 (-0.38, -0.35) | -0.35 (-0.40, -0.30) | -141.39 (-165.02, -116.97) | -2578.50 (-2680.00, -2474.97) | - |
| LightGBM | 0.11 (0.09, 0.14) | -0.42 (-0.43, -0.40) | -0.32 (-0.37, -0.27) | -128.56 (-152.00, -105.00) | -2929.70 (-3023.00, -2839.95) | - |
| Logistic Regression | 0.09 (0.07, 0.12) | -0.40 (-0.42, -0.39) | -0.35 (-0.40, -0.30) | -141.70 (-167.00, -117.00) | -2838.91 (-2934.03, -2744.98) | - |
| Random Forest | 0.12 (0.10, 0.15) | -0.42 (-0.43, -0.40) | -0.32 (-0.37, -0.26) | -127.07 (-151.00, -103.00) | -2931.50 (-3028.03, -2838.98) | - |
| XGBoost | 0.12 (0.10, 0.14) | -0.42 (-0.44, -0.41) | -0.34 (-0.39, -0.28) | -134.96 (-160.00, -112.00) | -2978.93 (-3071.05, -2890.00) | - |

Pooled performance measures for all models and audit filters for the expanding window with annual updates analysis. The performance differences are calculated by subtracting the corresponding model values with the audit filter performance values. ICI is not calculated for audit filters since they don't output prediction probabilities.
Definition of abbreviations: AUC = Area under the ROC Curve; FPR = False positive rate; ICI = Integrated calibration index; TP = True positive patients; FP = False positive patients.

Table S6: Performance difference between XGBoost and logistic regression.

| AUC | False positive rate | Sensitivity | True positives | False positives |
|---|---|---|---|---|
| **Performance differences in high sensitivity calibration** | | | | |
| 0.03 (0.01, 0.05) | -0.10 (-0.11, -0.09) | -0.04 (-0.07, -0.01) | -15.98 (-27.00, -6.00) | -689.53 (-760.00, -621.97) |
| **Performance difference in optimal calibration** | | | | |
| 0.03 (0.01, 0.05) | -0.02 (-0.03, -0.01) | 0.02 (-0.03, 0.06) | 6.74 (-11.02, 24.00) | -140.02 (-200.00, -79.97) |

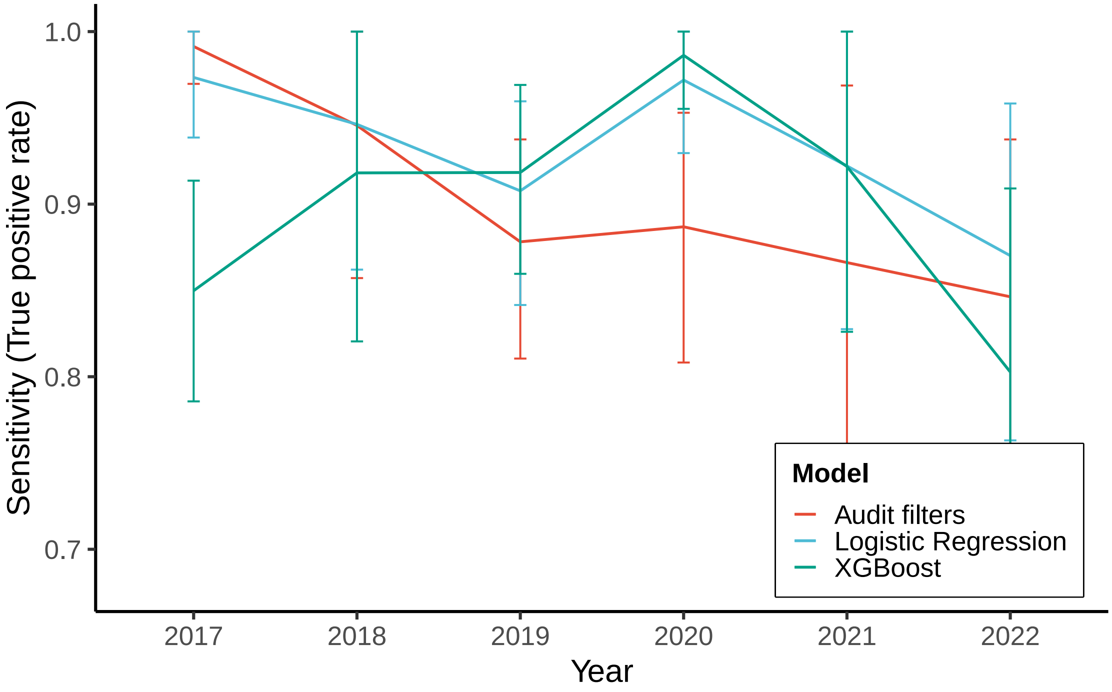


Figure S1: Annual true positive rates for years 2017 to 2022. Error bars indicate the 95% confidence intervall.

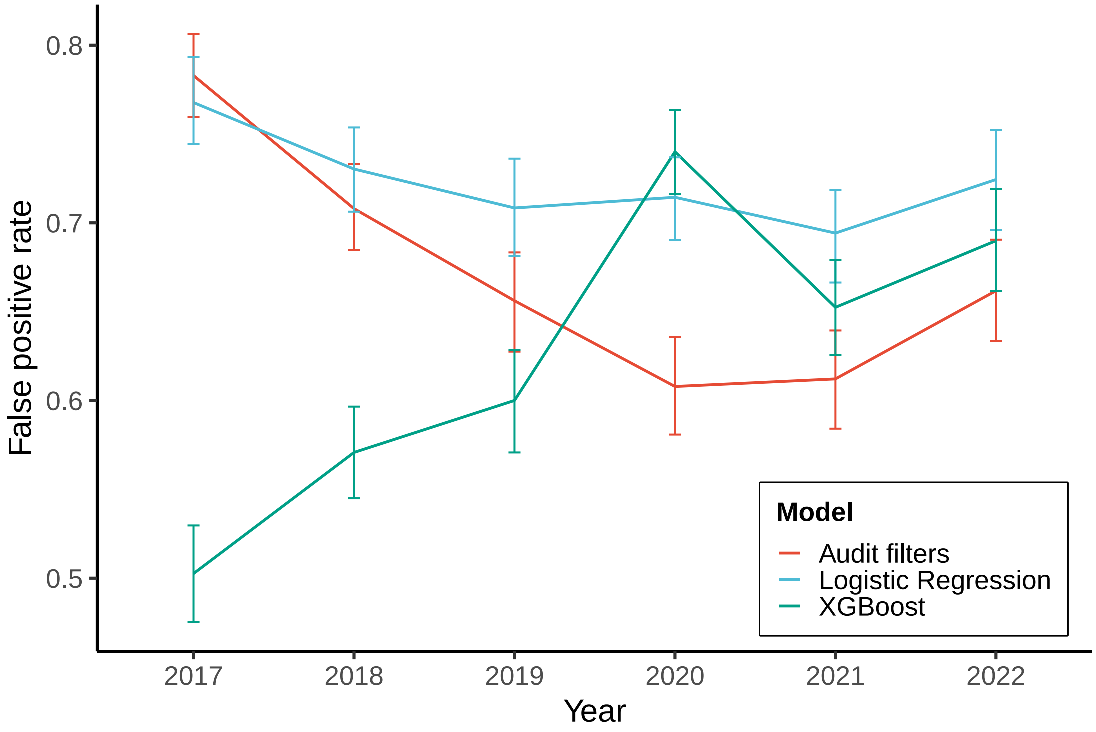


Figure S2: Annual false positive rates for years 2017 to 2022. Error bars indicate the 95% confidence intervall.

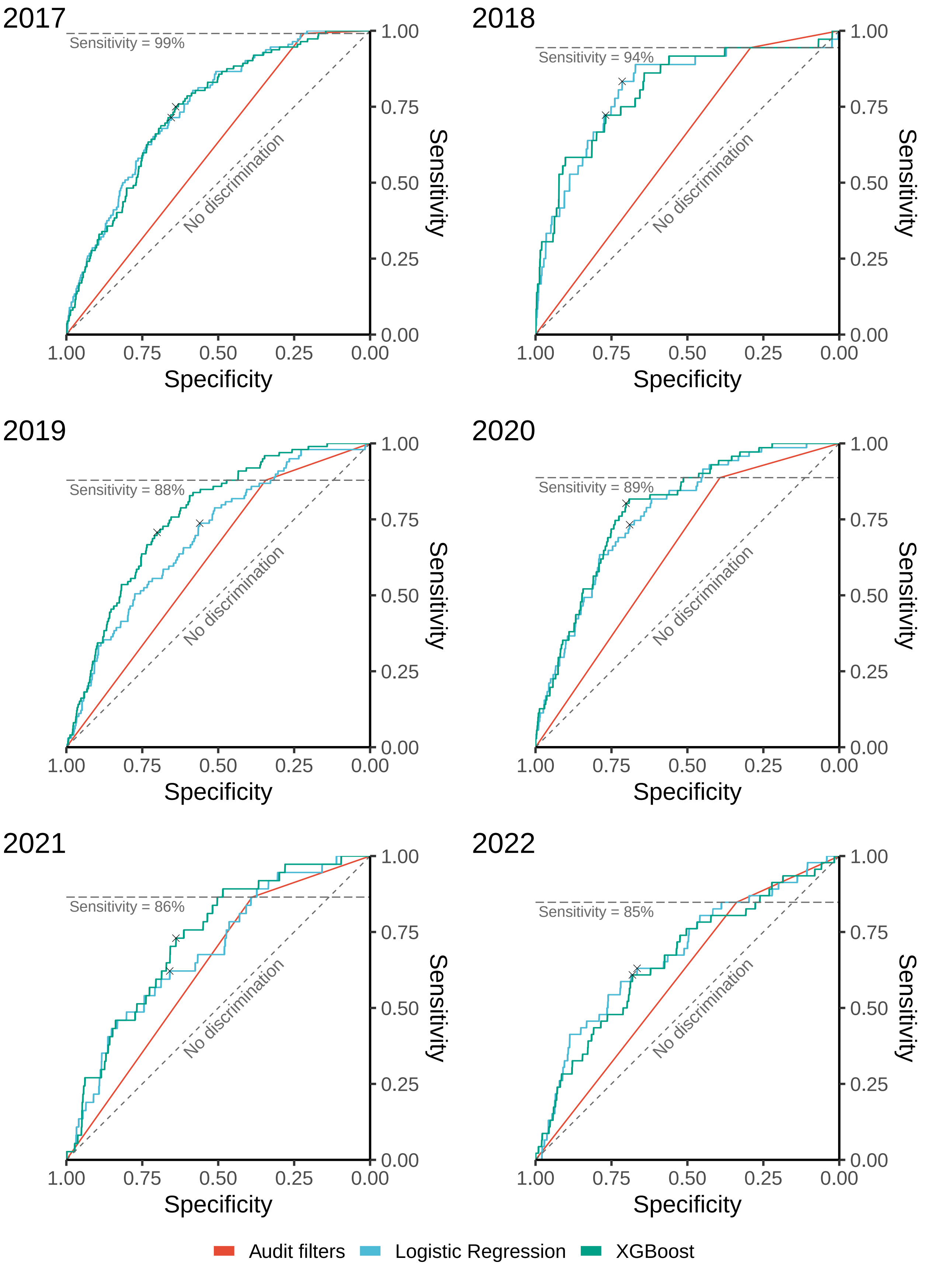


Figure S3: Receiver operating characteristic curves for years 2017-2022. Optimal calibration point is displayed as a cross on each respective model curve.
