# Supplementary material for: Predicting opportunities for improvement in trauma care using machine learning: a retrospective registry-based study at a major trauma centre
Source: BMJ Open. 2025 Jun 6;15(6):e099624. doi: 10.1136/bmjopen-2025-099624 (PMC12161345; doi:10.1136/bmjopen-2025-099624)
Supplement: Supplementary file 2 [file bmjopen-2025-099624.reviewer_comments.pdf]

PEER REVIEW HISTORY

BMJ Open publishes all reviews undertaken for accepted manuscripts. Reviewers are asked to complete a checklist review form and are provided with free text boxes to elaborate on their assessment. These free text comments are reproduced below.

ARTICLE DETAILS

Title (Provisional)

Predicting Opportunities for Improvement in Trauma Care Using Machine Learning: A Retrospective Registry-Based Study at a Major Trauma Centre

Authors

Attergrim, Jonatan; Szolnoky, Kelvin; Strömmer, Lovisa; Brattström, Olof; Whilke, Gunilla; Jacobsson, Martin; Gerdin Wärnberg, Martin

---

VERSION 1 - REVIEW

**Reviewer**	**1**
**Name**	**Moore, Lynne**
**Affiliation**	**CHU research center, pavillon Enfant-Jésus, Trauma, emergency medicine and intensive care**
**Date**	**24-Feb-2025**
**COI**	**None**

Thank you for the opportunity to review this manuscript. The idea of using AI based on various sources of readily available data to identify opportunities for improvement is very compelling. The use of TROPID-AI reporting guideline is appropriate. However, I have several major concerns:

1. My biggest concern is the audit filters used. These appear to be local audit filters and no information on how they were developed is given. Current practice to identify opportunities for improvement in most trauma systems is the use of quality indicators that measure adherence to CPG recommendations and are evidence-informed, consensus-derived and validated with trauma registry data. This does not seem to be the case for the audit filters proposed in this study, which are mostly non-specific (e.g. SBP<90 mmHg' or 'massive transfusion').

2. One potential disadvantage is the generic production of 'opportunities for improvement' without labelling the reason (i.e. the recommended process of care that were not adhered to) as process quality indicators do. Could the proposed method address this?

3. The authors state ‘Comparing these models to entire systems via a combination of quantitative screening and several human reviews, including a multidisciplinary review, is unfair and not the goal of this paper’. If the authors are referring to case review, I disagree. Identifying ‘opportunities for improvement’ is pointless unless cases are analyzed to assess if and how care could have been improved. To validate their model, case revision would be the next logical step.

4. Which framework was used for groupings of opportunities for improvement?

5. It seems understandable to me that given the simplicity of predictors, AI did not greatly outperform logistic regression. As I mentioned earlier, the true advantage of AI over simpler models will likely be shown using multiple linked data sources.

6. The treatment of missing data is not gold standard.

7. Table 2 – add a direct comparison of logistic regression compared to XGBoost

---

**Reviewer**	**2**
**Name**	**Uleberg, Oddvar**
**Affiliation**	**St. Olavs Hospital, Dept. of Emergency Medicine and Pre-hospital Services**
**Date**	**31-Mar-2025**
**COI**	**None**

Thank you for the opportunity to review the manuscript by Attergrim et al – “Predicting Opportunities for Improvement in Trauma Care: A registry-Based Cohort Study” (bmjopen-2025-099624). The manuscript addresses an important topic regarding the emerging use of machine learning models to establish clinical relevant prediction models.

In general I find the manuscript very well written, with only very few remarks. However, I must admit that the current description of the methods section is beyond my methodological competence and that the editor should seek more methodological expertise in order to provide relevant feedback to the authors. However, I have some remarks regarding other issues regarding the presentation.

Page 2 Lines 37-38: "trauma care quality improvement" is not an accepted MeSh term.

Page 3 Line 46: one space too much in front of the references

Page 4 Line 7/8: replace "has" with "have"

Page 4 Lines 20-21: these were to different databases/registries?

Page 5 Line 22: “different learners” relate to the different data models?

Page 10 Lines 9-11: you describe that the data models caused a reduction of false positives. Regarding the reduction of pure volume numbers, did the authors during the investigation

elaborate if this reduction was clinical relevant or simply observed this as a reduction based on the datamodelling?

The authors present a justified comparison of the different methods using AUC values/ROC curves. A valued addition for the more clinical related reader would be to add some simple descriptive numbers based on Supplemental File 1; how many patients fulfilled the mentioned criteria using audit filters versus what was the precision for the same criteria by using the investigated learners? This would be relevant when comparing the different methods. Would the actual differences be in pure numbers related to work load for the dedicated nurses.

VERSION 1 - AUTHOR RESPONSE

Comments From Reviewer 1

We thank Dr. Lynne Moore for the detailed and valuable review and comments. We have addressed each point below.

Comment 1: *My biggest concern is the audit filters used. These appear to be local audit filters and no information on how they were developed is given. Current practice to identify opportunities for improvement in most trauma systems is the use of quality indicators that measure adherence to CPG recommendations and are evidence-informed, consensus-derived and validated with trauma registry data. This does not seem to be the case for the audit filters proposed in this study, which are mostly non-specific (e.g. SBP<90 mmHg' or 'massive transfusion').*
Response: Thank you for highlighting this. We agree that more robust audit filters and quality indicators would have provided a stronger basis for the comparison, but we had to work with the audit filters that are currently being used. We have now clarified that we did not develop or implement audit filters as part of this study (p. 4), but assessed if the opportunities for improvement that were identified in the review process during which these audit filters were used could be predicted using machine learning. We now also acknowledge the lack of standardisation of the audit filters in our limitations (p. 12).

Comment 2: *One potential disadvantage is the generic production of 'opportunities for improvement' without labelling the reason (i.e. the recommended process of care that were not adhered to) as process quality indicators do. Could the proposed method address this?*
Response: The intended use of the proposed method is to identify patient cases for which an opportunity for improvement is likely. These cases should then be reviewed by humans to identify the recommended process of care that was not adhered to. So, the proposed method does not replace the case review, but provides a dynamic alternative to static audit filters. We have attempted to clarify this in the discussion/conclusion section (p. 12).

Comment 3: *The authors state 'Comparing these models to entire systems via a combination of quantitative screening and several human reviews, including a*

multidisciplinary review, is unfair and not the goal of this paper'. If the authors are referring to case review, I disagree. Identifying 'opportunities for improvement' is pointless unless cases are analyzed to assess if and how care could have been improved. To validate their model, case revision would be the next logical step.
Response: We agree that case review is essential for improving care. Just developing models to identify opportunities for improvement without discussing the cases at peer-review and implementing corrective measures serves little purpose. Our models are not intended to replace case review but to improve the initial screening process that is currently done using audit filters. The models serve as a more accurate "learnable" alternative to audit filters for identifying cases in need of peer-review. We have adjusted the aim to better reflect this purpose (p. 3).

Comment 4: *Which framework was used for groupings of opportunities for improvement?*
Response: In developing our groupings of opportunities for improvement we needed to take into consideration the chosen terminology from the local trauma quality database while still trying to maintain consistency with terminology used in previous published trauma quality literature. Our categorization draws directly from the categorization used in previously published work at our center analyzing possibly preventable deaths (1). We also incorporated elements from external trauma literature that used comparable definitions and classifications (2), to strive for alignment with the broader trauma care quality context.
We have changed the methodology in hope of better explaining our categorization (p. 4).

1. https://onlinelibrary-wiley-com.proxy.kib.ki.se/doi/10.1111/aas.13151
2. https://journals.lww.com/jtrauma/abstract/2007/12000/preventable_or_potentially_preventable_mortality.21.aspx

Comment 5: *It seems understandable to me that given the simplicity of predictors, AI did not greatly outperform logistic regression. As I mentioned earlier, the true advantage of AI over simpler models will likely be shown using multiple linked data sources.*
Response: This is a valuable point and something we ourselves have thought about. Even currently, there are more predictors we could have included, but we opted not to do so in order to keep 100% compliance with the variables in the Utstein Template for Uniform Reporting of Data following Major Trauma. This increases the applicability of our results in other settings. We have now updated the discussion section to reflect our thoughts on this based on this comment (p. 11).

Comment 6: *The treatment of missing data is not gold standard.*
Response: We agree that missing data was imputed in a suboptimal way. We have corrected the analysis to now use k-Nearest Neighbors for imputation. With this approach, we excluded the missing category for categorical variables and missing markers for continuous variables. The manuscript has been updated to reflect this (p. 5).

Comment 7: *Table 2 – add a direct comparison of logistic regression compared to XGBoost.*
Response: We agree that this is a valuable comparison to include in the manuscript. We have added it to Table S6 in the supplement to avoid overburdening Table 2 with additional analyses. We have referenced these results in the main text (p. 10).

Comments From Reviewer 2

We thank Dr. Oddvar Uleberg for their review and input regarding the presentation. We have adjusted the manuscript to address applicable comments.

Comment 4: *Page 4 Lines 20-21: these were to different databases/registries?*
Response: Yes, internally two different registries exist: the trauma registry and the trauma care quality database. The trauma registry is compliant with the Utstein Template for Uniform Reporting of Data following Major Trauma and is included in the national Swedish trauma registry (SweTrau). The trauma care quality database is an internal registry used for quality improvement and is not available in any national registry.

Comment 6: *Page 10 Lines 9-11: you describe that the data models caused a reduction of false positives. Regarding the reduction of pure volume numbers, did the authors during the investigation elaborate if this reduction was clinical relevant or simply observed this as a reduction based on the datamodelling?*
Response: This is a very relevant question, however, due to the current study being purely retrospective in nature, it is difficult to investigate whether the reduction in false positives, as presented in Table 2 and Table S5) is clinically relevant. We hope that a future clinical trial will examine this question.

Comment 7: *The authors present a justified comparison of the different methods using AUC values/ROC curves. A valued addition for the more clinical related reader would be to add some simple descriptive numbers based on Supplemental File 1; how many patients fulfilled the mentioned criteria using audit filters versus what was the precision for the same criteria by using the investigated learners? This would be relevant when comparing the different methods. Would the actual differences be in pure numbers related to work load for the dedicated nurses.*
Response: We agree that these are important numbers to include in order to estimate the possible clinical impact that the methodology could have. As pointed out, we have presented these numbers in the supplement. This was done to maintain focus on the primary performance metrics in the main text. We refer to and discuss these supplemental numbers in the third paragraph of the discussion (p. 11), as we agree these are very relevant figures. We hope this suffices.
